# Eicosapentaenoic Acid: between Cardiovascular Benefits and the Risk of Atrial Fibrillation

**DOI:** 10.2174/0118715303280825231122153024

**Published:** 2023-12-11

**Authors:** Filippo Egalini, Mattia Rossi, Mauro Massussi, Giulia Gaggero, Guglielmo Beccuti, Andrea Benso, Massimo F. Piepoli, Fabio Broglio

**Affiliations:** 1 Division of Endocrinology, Diabetes and Metabolism, Department of Medical Sciences, University of Turin, Corso Dogliotti 14, Turin, 10126, Italy;; 2 Cardiac Catheterization Laboratory and Cardiology, ASST Spedali Civili di Brescia, Department of Medical and Surgical Specialties, Radiological Sciences, and Public Health, University of Brescia, Brescia, Italy;; 3 Clinical Cardiology, IRCCS Policlinico San Donato, Piazza Malan, San Donato Milanese, 20097 Milan, Italy;; 4 Department of Biomedical Science for the Health, University of Milan, Via Festa del Perdono, 7, 20122, Milan, Italy

**Keywords:** Atrial fibrillation, cardiovascular events, eicosapentaenoic acid, omega-3, n-3 PUFA, EPA

## Abstract

In recent years, scientific research has increasingly focused on the cardiovascular benefits of omega-3 polyunsaturated fatty acids (n-3 PUFAs) supplements. The most promising results emerged from the new trials on a high-dose eicosapentaenoic acid (EPA)-only approach, instead of the previously prescribed therapy with EPA + docosahexaenoic acid (DHA). The evidence of the reduction of cardiovascular events in patients at high cardiovascular risk with EPA is intriguing. However, physicians have expressed concern about the potential high risk of atrial fibrillation (AF) occurrence due to such an approach. This study aims to investigate the current evidence on the cardiovascular benefits of EPA and its association with atrial arrhythmogenesis. Current guidelines consider EPA (as IPE) treatment for selected patients but with no specific indication regarding AF risk evaluation. We propose a flowchart that could be a starting point for the future development of an algorithm to help clinicians to prescribe EPA safely and effectively, especially in patients at high risk of incipient AF.

## 1 INTRODUCTION

Omega-3 polyunsaturated fatty acids (n-3PUFAs) are a family of fatty acids characterized by the closest double bond to the methyl end of the hydrocarbon chain to be on the third-to-last carbon. The most studied n-3PUFAs are represented by eicosapentaenoic acid (EPA; 20:5n-3) and docosahexaenoic acid (DHA; 22:6n-3), which play a pivotal role in cellular biology regulating cell phospholipidic membrane’s fluidity [1]. Moreover, n-3PUFAs are processed in prostaglandins, thromboxanes, and leukotrienes and are consequently involved in a wide range of biological processes [1]. The principal dietary source of EPA and DHA is fatty fish, also known as “oily fish”. Similarly, sea mammals, such as whales and seals, also are rich in EPA and DHA. N-3PUFAs can be found in several supplements: fish oils, cod liver oil, krill oil and some algal oils, and eventually in concentrated pharmaceutical-grade preparations of EPA and DHA, or EPA alone [2]. These compounds, particularly EPA alone, recently gained popularity due to the distinctive new evidence of a reduction in residual cardiovascular risk (RCVR), yet not without debate among experts [3, 4]. N-3PUFAs are generally considered safe drugs; the most reported adverse reactions include gastrointestinal symptoms, musculoskeletal pain, peripheral edema, gout, rash, bleeding, and atrial fibrillation (AF) [5].

Particularly, the augmented risk of AF has been outlined over the years, even recently, with conflicting results [6-11]. Definitive evidence on the causal role in AF of n-3PUFAs and their putative mechanisms is still lacking; in fact, it remains unclear whether this adverse event could be class- or molecule-specific or dose-dependent. To ensure an adequate evaluation of the risk/benefit ratio of n-3PUFAs treatment, an accurate analysis should be conducted. Our goal is, therefore, to investigate recent evidence for the cardiovascular benefit of EPA therapy and the risk of developing AF. Eventually, we propose a hypothetical flowchart as a base for subsequent studies aimed to formally define a clinical algorithm for safer and more effective prescription of EPA in patients at high risk of incipient AF.

## 2 EPA AND CARDIOVASCULAR RISK REDUCTION

Currently, the first-choice agents for secondary cardiovascular prevention are high-intensity statins, as recommended by major guidelines [3, 4]. They demonstrated the ability not only to reduce low-density lipoprotein cholesterol (LDLc) but also to exert a great reduction in both major adverse cardiovascular events (MACEs) (up to 40% reduction) and all-cause mortality (9% per 1.0 mmol/L of LDLc reduction) [12-15]. However, the scientific community has recently focused on effective ways of preventing consequences of the so-called residual cardiovascular risk (RCVR), *i.e*., the burden of incident vascular events or progression of established vascular damage persisting in patients treated with current evidence-based recommended care and specifically upon statin therapy [16]. Numerous epidemiologic and Mendelian randomization studies proved how triglycerides (TG) are a major prognostic factor for RCVR, even without being directly involved in plaque genesis [17-24]. However, neither traditional treatments for hypertriglyceridemia, such as niacin and fibrates, nor n-3 fatty acids initially showed the undeniable ability to reduce MACEs [25-29]. Even recently, the PROMINENT trial observed no difference in MACEs reduction between the placebo and pemafibrate groups, regardless of the higher decrease in TG reported in the treatment group. The authors selected a population of LDL-controlled patients with diabetes, older than 50 and/or with a history of cardiovascular disease (ASCVD). The primary endpoint included a composite of myocardial infarction (MI), ischemic stroke, any revascularization or death from cardiovascular causes [30]. Regarding n-3PUFAs, clinical trials before 2010, such as VITAL, GISSI-HF and ASCEND, could not show any effect of 1 gr/day of combinations of EPA and DHA on MACEs, even when revascularization was included in non-controlled LDL, primary prevention populations. EPA/DHA ratio was, respectively, 1.2, 0.83, and 1.2 [25, 26, 31]. In 2007, a Japanese open-label clinical trial named JELIS reported significantly reduced MACEs in hypercholesterolemic patients by adding 1.8 gr of EPA daily to a low-intensity statin therapy *versus* statin therapy alone. The primary endpoint was proved in the whole study population, regardless of whether patients were in primary or secondary cardiovascular prevention, with a hazard ratio (HR) of 0.81 (*p* = 0.011). However, at post-hoc analysis, significance was only reached when in secondary prevention [6]. Following JELIS example, the Reduction of Cardiovascular Events with Icosapent Ethyl–Intervention Trial (REDUCE-IT) was designed in 2017 to investigate the effect of treatment with 4 gr/day of Icosapent ethyl (IPE), a highly purified and stable EPA ethyl ester, in a population of over 8,000 hypertriglyceridemic, LDL-controlled patients on statins, with a history of established cardiovascular disease. The authors reported a significantly lower HR for both a composite endpoint of cardiovascular death, nonfatal MI, nonfatal stroke, coronary revascularization or unstable angina (HR = 0.75, *p* < 0.001), and a composite endpoint of cardiovascular death, nonfatal MI or nonfatal stroke (HR = 0.75, *p* = 0.044) [7]. Interestingly, in REDUCE-IT, the reduction of MACEs was not associated with a decrease in serum TG. These findings were later corroborated by the EVAPORATE study. Particularly, EVAPORATE evaluated cardiovascular risk (CVR) in terms of progression rates of low attenuation plaque instead of cardiovascular events in an identical population and demonstrated significant plaque stabilization after 18 months of treatment with IPE 4 gr/day. The authors observed a reduction in low attenuation plaque volume by 17% in the treatment group *versus* an increase of 109% in the placebo group (*p* = 0.006) [32]. Furthermore, the EPA effect on MACEs was confirmed in several specific subpopulations, such as patients with prior MI, diabetes or renal function impairment, throughout post-hoc analysis [33, 34]. A summary of the results of these trials is represented in Table **1**. Conversely, in recent trials, the combination of EPA and DHA did not show the same results. For instance, the OMEMI trial (Omega-3 Fatty acids in Elderly with Myocardial Infarction) was an investigator-initiated, multicenter, randomized clinical trial administering 1.8 g n-3 PUFA (0.93 gr EPA + 0.66 gr DHA) *versus* placebo (corn oil) daily in secondary-prevention patients aged 70 to 82 years with recent (2-8 weeks) acute MI. No significant difference in MACEs was shown between patients on n-3 PUFA and placebo (*p* = 0.60) [35]. Even STRENGTH trial failed to prove a reduction in MACEs in a REDUCE-IT-like population of high-risk, statin-treated, LDL-controlled, hypertriglyceridemic patients. They were treated with a combination of EPA and DHA, 4 gr/day. EPA/DHA ratio was 1,2 [8]. A conclusive explanation regarding such a concrete discrepancy in results between these studies, mainly REDUCE-IT and STRENGTH, is still lacking. Comparison is, in fact, made difficult because of the large heterogeneity of protocols, leaving uncertainty about issues, such as daily dose, composition (EPA *versus* EPA+DHA), and EPA/DHA ratio [36]. For instance, some authors called for caution regarding the results of REDUCE-IT, criticizing the paradoxical apparent absence of the effect of EPA on anti-inflammatory and lipid-related biomarkers, such as TG, LDL, high-sensitivity C reactive protein (hsCRP) [37]. Conversely, evidence was gathered on how the same markers were altered (respectively +2.2%, +10.9%, and +32.3%) in the placebo group, leaving space for a possible detrimental role of the substance implemented as placebo (mineral oil), as an explanation for the unexpected magnitude of the result [36]. This was also confirmed when Doi *et al*. selected two samples of patients mimicking the populations of REDUCE-IT and STRENGTH and studied the effect of both active (EPA ± DHA) and placebo (mineral and corn) oils on events and the other biomarkers [38]. Moreover, mineral oil has a laxative effect, so it could have possibly interfered with the absorption of other hypolipidemic treatments, such as statins, acting as a source of bias [36]. Some researchers differ, relying on the extensive, historical, safe prescription of mineral oil in both clinical and research contexts [39-41]. In addition, Lakshmanan *et al.* reported no significant differences in the progression of total plaque and total non-calcified plaque volume by coronary CTA in mineral oil placebo participants compared to non-mineral oil placebo participants drawn from two different randomized placebo-controlled trials [42]. Finally, a possible rise in CVR determined by mineral oil was estimated to be around 3%, which was argued by experts not to be sufficient to justify the large benefit of EPA proven in REDUCE-IT [43-46]. They hypothesize the possibility of a positive EPA-specific action and/or a detrimental role of the combination EPA+DHA. This aspect is still a source of large debate in the scientific community. Considering all these data, the 2021 European Society of Cardiology (ESC) and the 2019 European Atherosclerosis Society (EAS) guidelines proposed to consider the prescription of EPA (as IPE) 4 gr/day in subjects with established cardiovascular disease with TG between 150 to 499 mg/dl despite statin treatment and instead of the previously common use of 1-2 gr/day of the combination EPA+DHA [3, 4]. Finally, the most recent trial on this topic was presented on November 6^th^, 2022, during the American Heart Association’s congress in Chicago: the Randomized Trial for Evaluation in Secondary Prevention Efficacy of Combination Therapy–Statin and Eicosapentaenoic Acid (RESPECT-EPA). In this trial, 2,506 patients with a history of ASCVD and an EPA/Arachidonic Acid (AA) ratio lower than 0.4 were randomized to either EPA 1.8 gr/day or standard-statin therapy (control group) [47]. A significant reduction in events was found only for their secondary endpoint [*p* = 0.0306], while the primary endpoint group [*p* = 0.05^47^] reached statistical significance only after an interesting yet convoluted, post-hoc analysis [*p* = 0.0202] [48]. This, together with the fact that the authors themselves acknowledged that the study could be underpowered, calls for caution, and final results should be waited before any conclusion is drawn [49]. In conclusion, a high-dose EPA-only approach could represent a promising line of therapy for RCVD reduction. Yet, definitive evidence is still lacking. The contradictory outcomes of REDUCE-IT and STRENGTH challenge the undeniability of EPA-specific benefits. In fact, if this gain could reflect a peculiar effect of the EPA-alone *versus* EPA+DHA approach, the possible causality role and weight of mineral oil should find definitive exclusion. However, if confirmed, the discrepancy between the results of old trials [6, 25, 26, 31] and more recent ones seems to be related to the difference between selected populations, respectively, non-controlled LDL primary prevention *versus* LDL-controlled secondary prevention. This could be interpreted as both a limit, in terms of selection bias, and a virtue because it shifted the focus to a specific subgroup of patients, in which RCVR could be effectively targeted by EPA. Furthermore, it is crucial to the lack of correlation between the TG-lowering effect of EPA and its CVR impact. In fact, hypertriglyceridemia seems to be only an indicator for a better selection of treatable subjects and not the target of our intervention.

## 3 EPA AND AF: BENEFITS AND HARM

The new evidence on a beneficial effect on the risk of atherosclerotic cardiovascular events of EPA has raised concerns on the possible related adverse effects of this molecule, such as AF. Interestingly, pre-clinical studies outlined a possible protective role in AF pathogenesis for n-3PUFAs supplements. They demonstrated increased cardiomyocyte membrane stability and fluidity to modulate AF triggering channels and induce an anti-inflammatory, anti-thrombotic, and anti-fibrotic effect [50]. On the contrary, researchers failed to confirm this AF-protective role in a clinical setting. They witnessed an increased risk of AF associated with n-3PUFAs treatment, leaving more questions than answers [7–11]. Currently, AF is a known adverse event of the therapy with n-3PUFAs, although the mechanistic basis of the net arrhythmogenic effect of these drugs remains unclear. Experimental studies focused mainly on myocardial electrophysiological properties reported that n-3PUFAs alter the balance of ionic cardiac currents, causing a variation in ventricular action potential duration [51]. Conversely, less is known about how n-3PUFAs affect heart atria, with recently published data suggesting that not all of them behave similarly. Cell membrane lipids influence the conformational rearrangements that lead to ion channels opening and closure. Differently from EPA, DHA leads to a gain-of-function of the newly discovered mechanosensitive ion channel PIEZO1, which is a large mechanotransducer crucial for initiating mechano-chemo transduction [52]. Therefore, a DHA-predominant environment results in an increased influx of calcium and other cations. Collectively, these changes lead to a longer potential duration and promote calcium-dependent signaling, and consequently, in delayed afterdepolarizations that trigger AF. EPA seems, conversely, to fasten the inactivation and, consequently, the closure of the channel [52-54]. Moreover, EPA can be beneficial after a triggering stimulus of AF, as reported in animal models, and it might prevent atrial electrical remodeling [55, 56]. Parallel to the encouraging results obtained in experimental settings, various studies aimed to explore the anti-arrhythmogenic impact of EPA supplements on cardiac patients. Suenari *et al*. hypothesized that EPA reduces pulmonary vein arrhythmogenesis through the mechanoelectrical feedback generated by nitric oxide (NO) production. This effect seems to be thwarted by the presence of inhibitors of NO synthase or omega-9 fatty acids (*e.g*., oleic acid) [57]. The above-cited hypothesized mechanisms of n-3PUFAs on atrial arrhythmogenesis are summarized in Fig. (**1**). Finally, an intriguing analysis from the Swiss Atrial Fibrillation study showed that higher blood levels of EPA in patients with AF correlate inversely with the prevalence of ischemic brain infarctions [58]. However, as previously mentioned, cardiovascular outcome trials on n-3 PUFAs (both EPA only and EPA+DHA regimens) outlined an augmented risk of AF with this treatment. This effect might be driven by the total daily dose of n-3PUFAs. In the OMEMI trial, elderly patients were given 1.8 gr/day of EPA+DHA, and there was no significant difference in new-onset AF between the n-3PUFAs group and placebo group (7.2% *vs*. 4%, HR = 1.84, *p* = 0.06) [35]. Referring to high n-3PUFAs dose clinical investigations, the STRENGTH trial reported new-onset AF in 2.2% of patients in the experimental arm (EPA+DHA) *versus* 1.3% in the placebo arm (HR = 1.69, *p* < 0.001) [8]. Also, the REDUCE-IT trial recorded new onset or worsening of AF in 5.3% of those receiving IPE and in 3.9% of the placebo group (HR = 1.35, *p* = 0.003) [7]. Furthermore, the available information regarding safety in the low-EPA dose RESPECT-EPA trial reported a higher rate of AF-onset in the purified EPA group (3.1% *vs*. 1.6%, *p* = 0.017) [50].

Comparing novel and previous studies, it emerged that the HR of AF per 1 gr/day increase in n-3PUFAs supplements was 1.11 [59]. The VITAL trial was designed to shed light on the potential effect of the n-3PUFAs therapy to raise incident AF in a large population without prior cardiovascular disease. Treatment with EPA+DHA compared to the placebo did not result significantly different in terms of AF occurrence; the HR of a 1 gr/day dosage of EPA+DHA was 1.09 (*p* = 0.19) [9]. Currently, there are no large studies designed to investigate AF with EPA+DHA or EPA-only treatment. However, there has been speculation on the possible different impact of EPA *versus* the combination of EPA+DHA on the risk of new-onset AF. Indeed, comparing the results of REDUCE-IT and STRENGTH, single EPA therapy seems to expose patients to a lower risk of AF than treatment with EPA+DHA. Nevertheless, the heterogeneity of the population of the two trials brings the need for further evidence. Overall, these data suggest that the cardiovascular effects of increasing n-3PUFA levels through supplements are complex, involving both potential benefits and harm.

## 4 MANAGING EPA THERAPY: A PRACTICAL FLOWCHART TO BALANCE CARDIOVASCULAR RISK REDUCTION AND AF OCCURRENCE

**Fig. (2):**
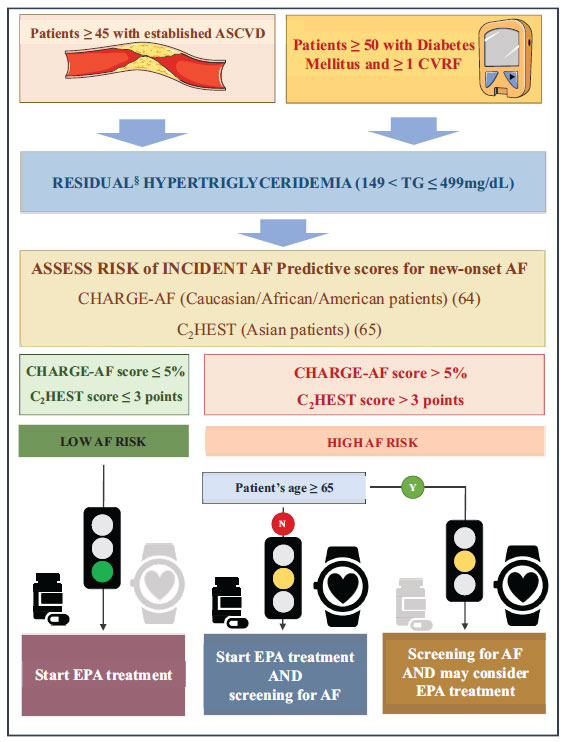
A flowchart to guide EPA prescription, considering AF risk. EPA: Eicosapentaenoic acid, ASCVD: atherosclerotic cardiovascular disease, CVRF: cardiovascular risk factor, AF: atrial fibrillation, 4 gr/day (high-dose regimen), according to the current guidelines § Despite maximally tolerated statin treatment.

Current guidelines recommend the prescription of EPA (as IPE) in high-CVR patients with hypertriglyceridemiawithout mentioning any screening for AF [4]. Established risk factors for AF include increasing age, male gender, diabetes, obesity, MI, heart failure, hypertension, cigarette smoking, and alcohol abuse [60]. Past and recent trials focused on the cardiovascular preventive role of n-3PUFAs shared a relatively similar design and enrolled patients at high cardiovascular risk with a greater prevalence of the above-cited conditions acting as confounding factors for new-onset AF. However, the reported incidence of AF was very low, even in high-dose n-3PUFA trials. There was a slightly reduced risk in patients receiving EPA compared to those taking EPA+DHA, as mentioned in Table **1**. Recently, experts advocated the need for a careful prescription of n-3PUFAs in individuals susceptible to developing AF [61]. In this light, we propose a flowchart for safer management of EPA therapy, especially in patients at high risk of incident AF (Fig. **2**).

Our strategy can be implemented in a specific subset of hypertriglyceridemic, high-cardiovascular-risk patients with established atherosclerotic cardiovascular disease (ASCVD) and/or patients with diabetes mellitus with severe target organ damage. This is because they seem to benefit the most from this therapy, as shown in the aforementioned trials [7, 32]. Included patients should have TG values between 150 and 499 mg/dL since data on MACEs for TG ≥ 500 mg/dL is still lacking, while minor evidence exists on IPE-related reduction in large VLDL, total LDL, small LDL, and total HDL particle concentrations and VLDL particle size [62]. EPA regimen should consist of 4 gr/day (high-dose regimen), according to the current guidelines [3, 4]. Notably, patients with established ASCVD and/or patients with diabetes with severe target organ damage seem to benefit the most from this therapy.

Conversely, elderly patients, specifically those older than 65, may have lower benefits from EPA, as shown by subgroup analyses of the REDUCE-IT trial [7]. Moreover, increasing age is a major risk factor for AF. As indicated in our flowchart, it would be advisable to carefully consider whether to start treatment with EPA in older patients. CHARGE-AF score and CH2EST score are two simple and validated models able to predict the risk of incident AF in several populations [63, 64]. When EPA is prescribed to patients at high risk for incident AF, we advocate for systematic screening of atrial arrhythmia as a means to prevent AF-related morbidity and atrial remodeling [65]. While novel screening technologies, such as smartphone applications and smartwatches, have demonstrated significantly higher sensitivity and specificity compared to pulse-taking screening and random 24-hour Holter monitoring, it is important to emphasize that AF diagnosis should always be confirmed with an ECG. While the innovative screening tools offer promising advancements in detecting AF, they should be considered as initial screening methods rather than definitive diagnostic tools. Subsequent confirmation with an ECG allows for accurate and reliable diagnosis, enabling appropriate management and treatment decisions [66, 67]. On the other hand, patients at low risk for incident AF do not take advantage of systematic AF screening. Nonetheless, every patient treated with EPA should be aware of AF symptoms to seek appropriate cardiological care if necessary.

## CONCLUSION

The striking evidence of a potential reduction in cardiovascular events in patients at high CVR with EPA is intriguing, despite the need for both further confirmation and a better understanding of the mechanisms of these beneficial effects. Definite subgroups of patients with established cardiovascular disease and residual hypertriglyceridemia might benefit the most from EPA. Despite physicians’ concern about the risk of AF, studies reported that n-3PUFAs-related AF risk is low, even though few data are available on an EPA-only regime. Results from large randomized controlled trials are needed to establish the actual magnitude of AF risk due to EPA therapy and if it is smaller than the risk of AF in individuals receiving EPA+DHA, as preliminary data seem to point out. We propose a flowchart as a starting point for further studies focused on developing an algorithm to help physicians manage this new promising therapy safely and effectively. We believe that a validated algorithm could be very useful for a more conscious prescription of EPA, especially in the subgroup of patients intrinsically at high risk of developing AF, such as people older than 65, in which the cardiovascular benefit from EPA seems to be lower.

## Figures and Tables

**Fig. (1):**
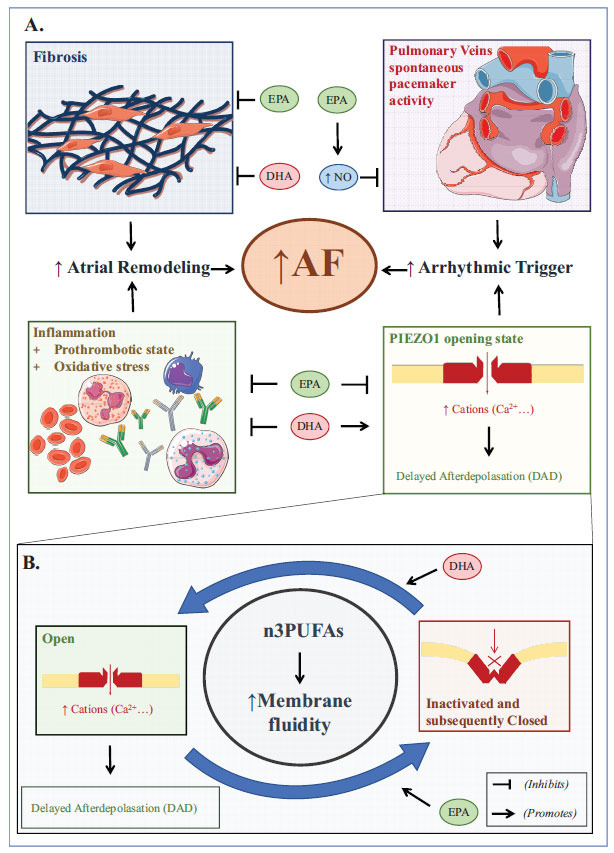
Possible mechanisms of benefits and harms of n-3PUFAs on atrial arrhythmogenesis (**A**) and the newly discovered role of EPA on PIEZO1 ion channel (**B**).

**Table 1:** Summary of the results of main trials on the cardiovascular effect of n-3PUFAs.

**-**	**Sample Size**	**Intervention**	**Control**	**Lipid Profile at Enrollment**	**Other** **Antihyperlipidemic Treatment at Study Entry**	**CVR Outcome**	**Pre-Existing AF**	**AF Related Events:** **n-3PUFAs *vs* Placebo**
VITAL* [9, 25]	25’871	840 mg/day n-3PUFAs (460 mg EPA + 380 mg DHA)	Olive oil	Not reported	Cholesterol-lowering unspecified therapy in comparable percentage in both arms. At a subgroup analysis, baseline statin therapy did not prove significantly different outcomes.	Primary endpoint: - MACEs^§^, HR = 0.92 (0.80 - 1.06), *p* = 0.24 Secondary endpoints: - Total MI, HR = 0.72 (0.59 - 0.90), p NR - Cardiovascular Death, HR = 0.96 (0.76 - 1.21), p NR - Total stroke, HR = 1.04 (0.83 - 1.31), p NR - Expanded MACEs^§^, HR = 0.93 (0.82 - 1.04), p NR	Excluded in pre-specified subsequent analysis (- 752 patients)	469 (3.7%) *vs* 431 (3.4%), HR = 1.09, (0.96 - 1.24) *p* = 0.19
ASCEND* [10, 31]	15’325	840 gr/day n-3PUFAs (460 mg EPA + 380 mg DHA)	Olive oil	Total C: - < 155 mg/dL: 4’554 (30%) - 155-193 mg/dL: 3’795 (24%) - ≥ 193 mg/dL. 1’470 (10%) - Non available: 5’661 (36%) Non-HDLc - < 97 mg/dL 3’390 (22%) - 97-135 mg/dL: 4’393 (28%) - ≥ 135 mg/dL 2’017 (13%) - Not available: 5’680 (37%) TG: - Not measured	Population description reports statins (comparable percentage in both arms). Other antihyperlipidemic drugs not specified.	Primary endpoint: - First serious vascular event^§^: Rate ratio = 0.97 (0.87 - 1.08), *p* = 0.55 Secondary endpoint: - Serious vascular event or revascularization^§^: Rate ratio = 1.00 (0.91 - 1.09), *p* = NR	Excluded in pre-specified subsequent analysis (- 155 patients)	1177 total AF events, (7.7%) *vs* (7,6%), HR = 1.02 (0.91 - 1.15), P=NR
GISSI-HF* [11, 26]	6’975	850-882 mg/day n-3PUFAs (EPA+DHA in 1:1.2 rate)	Olive oil	Total C (available for 6’918 patients): - ≤ 188 mg/dL: 3’467 (50%) - > 188 mg/dL: 3’451 (50%)	Patients without contraindications to statins were randomly assigned to Rosuvastatin 10 mg. Population description reports comparable percentage in both arms. Other antihyperlipidemic drugs not specified.	Secondary endpoints: - Cardiovascular Death, HR = 0.90 (0.81 - 0.99), *p* = 0.045 - Fatal and non-fatal MI, HR = 0.82 (0.63–1.06), *p* = 0.12 - Fatal and non-fatal Stroke, HR = 1.16 (0.89–1.51), *p* = 0.27	Excluded in pre-specified subsequent analysis (- 2’534 patients)	241 (11.0%) *vs* 202 (9.0%), HR = 1.23 (0.96 - 1.25), *p* = 0.03
JELIS [6]	18’645	1.8 gr/day EPA (> 98% purified ethyl ester)	Statins alone (non-blinded)	TotalC, EPA group - mean 7.11 mmol/L (SD: 0.67) TotalC, control group - mean 7.11 mmol/L (SD: 0.68) LDLc, EPA group - mean 4.69 mmol/L (SD: 0.76) LDLc, control group - mean 4.70 mmol/L (SD: 0.75) HDLc, EPA group - mean 1.52 mmol/L (SD: 0.46) HDLc, control group - mean 1.51 mmol/L (SD: 0.44) TG, EPA group - median 1.73 mmol/L (IQR: 1.23 - 2.48) TG, control group - median 1.74 mmol/L (IQR: 1.25 - 2.49)	Pravastatin 10 mg/day or Simvastatin 5 mg/day. If uncontrolled hypercholesterolemia: Pravastatin 20 mg/day or Simvastatin 10 mg/day.	Primary endpoints: - All population MACEs^§^, HR = 0 0.81 (0.69–0.95), *p* = 0.01 - I^ary^ prevention MACEs^§^, HR = 0.82 (0.63–1.06), *p* = 0.13 - II^ary^ prevention MACEs^§^, HR = 0.81 (0.66–0.998), *p* = 0.048	AF not evaluated	AF not evaluated
REDUCE-IT [7]	8’179	4 capsules/day each containing 998 mg of Icosapent ethyl (highly purified EPA ethyl ester)	Mineral oil	TG: - Inclusion-criteria range: 150-499 mg/dL, - median 216 mg/dL, LDLc: - Inclusion-criteria range: 40-100 mg/dL, - - median 75 mg/dL.	Stable dose of a statin for at least 4 weeks.	Primary endpoint: - MACEs^§^, HR = 0.75 (0.68 - 0.83), *p* < 0.001 Secondary endpoint: - Core MACEs^§^. HR = 0.74 (0.65 - 0.83), *p* < 0.001	Not excluded	New-onset AF(non-adjudicated): 215(5.3%) *vs* 159(3.9%), HR = 1.35, *p* = 0.003; AF Hospitalization (adjudicated): (3.1%) *vs* (2.1%), *p* = 0.004
STRENGTH [8]	13’078	4 capsules day each containing “at least 850 mg of n-3 PUFAs (mainly EPA+DHA)”	Corn oil	LDLc: - Inclusion criteria: < 100 mg/dL (or more if maximum tolerated dose of statin was reached); - median n-3PUFAs arm 75 mg/dL (IQR. 56-99) - median Placebo arm 75 mg/dL (IQR. 56-99) TG: - Inclusion-criteria range: 180-500 mg/dL, - median n-3PUFAs arm: 239 mg/dL (192 - 307) - median Placebo arm: 240 mg/dL (191 - 309)	Statin for at least 4 weeks.	Primary endpoints: - Primary MACEs^§^ (total population), HR = 0.99 (0.90 - 1.09), *p* = 0.84 - Core MACEs^§^, HR = 1.05 (0.93 - 1.19), p NR	Excluded	144 (2.2%) *vs* 86 (1.3%) HR = 1.69, *p* < 0.001.
OMEMI [35]	1,014	1.8 gr/day n-3 PUFA (930 mg EPA + 660 mg DHA)	Corn oil	LDLc, n-3PUFAs group - mean 75.1 mg/dL (SD: 25.9) LDLc, control group - mean 77.0 mg/dL (SD: 26.1) HDLc, n-3PUFAs group - mean 49.3 mg/dL (SD: 15.2) HDLc, control group - mean 49.8 mg/dL (SD: 15.2) TG, n-3PUFAs group - mean 115.4 mg/dL (SD: 72.1) TG, control group - mean 107.4 mg/dL (SD: 29.5)	Population description reports statins (comparable percentage in both arms).	Primary outcome - Composite MACEs^§^, HR = 1.07 (0.82 – 1.40), p NR - Death as first event, HR = 1.01 (0.54 – 1.88), p NR - Nonfatal acute myocardial infarction, HR = 1.14 (0.72 – 1.80), p NR - Stroke, HR = 1.37 (0.65 – 2.88), p NR - Unscheduled revascularization, HR = 0.66 (0.34 – 1.30), p NR - Hospitalization for heart failure, HR = 1.19 (0.62 – 2.26), p NR - All-cause mortality, HR = 1.01 (0.60 – 1.71), p NR	Escluded (n-3 PUFA, n. 372; placebo, n. 387)	28 (7.2%) *vs* 15 (4.0%), HR = 1.84 (0.98–3.45), *p* = 0.06
EVAPORATE [32]	68	4 capsules/day each containing 998 mg of Icosapent ethyl (highly purified EPA ethyl ester)	Mineral Oil	TG: - Inclusion-criteria range: 135–499 mg/dL, - mean 259.1±78.1 mg/dL, LDLc: - Inclusion-criteria range: 40-115 mg/dL, - mean value non reported.	On stable statin therapy, ± ezetimibe, diet and exercise for ≥ 4 weeks.	Primary outcome: - Low Attenuation Atherosclerotic Plaque reduction: - 0.3 ± 1.5 *vs*. 0.9 ± 1.7 mm3, *p*.0.006.	AF not evaluated	AF not evaluated
RESPECT-EPA [49]	2’460	1.8 gr/day Icosapent ethyl (highly purified EPA ethyl ester)	Statins alone (non-blinded)	TotalC, EPA group - median 159 mg/dL (IQR: 142 - 178) TotalC, control group - median 158 mg/dL (IQR: 140 - 176) LDLc, EPA group - median 80.6 mg/dL (IQR: 67.4 - 97.1) LDLc, control group - median 80.6 mg/dL (IQR: 65.8 - 95.8) HDLc, EPA group - median 49 mg/dL (IQR: 41 - 58) HDLc, EPA group - median 49 mg/dL (IQR: 42 - 58) TG, EPA group - median 120 mg/dL (IQR: 86 - 173) TG, control group - median 117 mg/dL (IQR: 86 - 163)	Statin for at least 1 month.	Primary endpoint: - MACEs^§^, HR = 0.785 (0.616 - 1.001), *p* = 0.0547 Secondary endpoint: - MACEs (subset) ^§^. HR = 0.734 (0.554 - 0.973), *p* = 0.0306	Still not available	38 (3.1%) *vs* 20 (1.6%) *p* = 0.017

**Abbreviations:** AF: Atrial Fibrillation, CVR: cardiovascular risk, MACEs: Major Cardiovascular Events, MI: Myocardial Infarction, EPA: Eicosapentaenoic Acid, DHA: Docosahexaenoic Acid, n-3PUFAs: n-3Polyunsaturated Fatty Acids, IQR: InterQuartile Range, SD: standard deviation, LDLc: Low-density-lipoprotein cholesterol, TG: Triglycerides, Total C: Total Cholesterol, HDLc: High-density-lipoprotein cholesterol, Non HDLc: Non-high-density-lipoprotein cholesterol [=TotalC - HDLc], p.NR: non-reported p-value. P-values that reached statistical significance are reported in italics. * For these studies, data on AF were reported separately in specific subsequent analyses. § MACEs were defined differently in studies; for more detail, please refer to text or references.

## LIST OF ABBREVIATIONS

AF: Atrial Fibrillation
CVR: Cardiovascular Risk
DHA: Docosahexaenoic Acid
EPA: Eicosapentaenoic Acid
HR: Hazard Ratio
n-3PUFAs: Omega-3 Polyunsaturated Fatty Acids
RCVR: Residual Cardiovascular Risk
hsCRP: High-sensitivity C Reactive Protein
